# Application of Advanced Mass Spectrometry-Based Proteomics to Study Hypoxia Driven Cancer Progression

**DOI:** 10.3389/fonc.2021.559822

**Published:** 2021-02-23

**Authors:** Arada Vinaiphat, Jee Keem Low, Kheng Wei Yeoh, Wee Joo Chng, Siu Kwan Sze

**Affiliations:** ^1^ School of Biological Sciences, Nanyang Technological University, Singapore, Singapore; ^2^ Department of Surgery, Tan Tock Seng Hospital, Singapore, Singapore; ^3^ Department of Radiation Oncology, National Cancer Centre Singapore, Singapore, Singapore; ^4^ Department of Hematology-Oncology, National University Cancer Institute, National University Health System, Singapore, Singapore

**Keywords:** cancer, hypoxia, proteomics, mass spectrometry, SILAC, iTRAQ, TMT, MRM

## Abstract

Cancer is one of the largest contributors to the burden of chronic disease in the world and is the second leading cause of death globally. It is associated with episodes of low-oxygen stress (hypoxia or ischemia/reperfusion) that promotes cancer progression and therapeutic resistance. Efforts have been made in the past using traditional proteomic approaches to decipher oxygen deprivation stress-related mechanisms of the disease initiation and progression and to identify key proteins as a therapeutic target for the treatment and prevention. Despite the potential benefits of proteomic in translational research for the discovery of new drugs, the therapeutic outcome with this approach has not met expectations in clinical trials. This is mainly due to the disease complexity which possess a multifaceted molecular pathology. Therefore, novel strategies to identify and characterize clinically important sets of modulators and molecular events for multi-target drug discovery are needed. Here, we review important past and current studies on proteomics in cancer with an emphasis on recent pioneered labeling approaches in mass spectrometry (MS)-based systematic quantitative analysis to improve clinical success. We also discuss the results of the selected innovative publications that integrate advanced proteomic technologies (*e.g.* MALDI-MSI, pSILAC/SILAC/iTRAQ/TMT-LC-MS/MS, MRM-MS) for comprehensive analysis of proteome dynamics in different biosystems, including cell type, cell species, and subcellular proteome (*i.e.* secretome and chromatome). Finally, we discuss the future direction and challenges in the application of these technological advancements in mass spectrometry within the context of cancer and hypoxia.

## Introduction

The global burden of cancer is substantial with millions of lives being lost each year (1, 2). In 2018, there were 18.1 million new diagnoses and 9.6 million deaths due to it (2). In addition, it also inflicts considerable morbidity to those affected both physically and psychologically. Advances in medical sciences have been tremendous with improvement in disease prevention, diagnosis, screening and treatment resulting in a decline in death rates for certain cancers (1, 2). For example, the development of the human papillomavirus (HPV) vaccine and the Papanicolaou (PAP) smear test for early detection of cervical cancer has decreased the death rates in the last half-century, and may even eliminate this cancer in the coming years (3–5).

Despite the improved understanding of cancer pathology and the remarkable leaps made in cancer treatment with a move towards personalized approaches, there remains challenges in current treatment approaches to eradicate and control cancer (6). Cancer is a complex disease that can no longer be understood as a simple enumeration of identical cancerous cells. Genetic diversity between different types of tumors and different patients makes it exceedingly difficult to eliminate all cancer cell types using a single therapeutic strategy (7–9). More importantly, recent studies using single-cancer-cell DNA sequencing to elucidate genetic diversification have revealed the heterogeneity among cancer cell populations (10–13). Heterogeneous cancer cells within a tumor, when subjected to selective pressures (Darwinian’s law of natural selection) either from treatment (chemotherapy, radiotherapy and biologicals) or unfavorable microenvironmental conditions such as hypoxia, results in the adaptation and selection of subclones with improved survival and malignant potential; with ensuing therapeutic resistance clinically (7, 9).

To overcome the limitations of current therapeutic approaches, we need to identify molecular signatures of malignant cell transformation and understand the carcinogenic processes involved. This will enable us to explicitly pinpoint precise targets for intervention as well as identifying novel biomarkers to monitor cancer progression and treatment response. While most advances in treatment have been based on genetic determinants (6), how these genetic changes translate to proteomic and phenotypic characteristics has not been well characterized; thus opening up opportunities for research in the field of proteomics (14). We will examine some of these advances with a focus on hypoxia which plays a central role in cancer progression and treatment resistance.

## Hypoxia-Driven Cancer and Hypoxia-Targeted Therapy

As aforementioned, the genetic diversity across the same and different types of tumors imposes challenges in finding the therapeutic targets that are unique to all cancerous cells, thus limiting the effectiveness of most cancer drugs. To worsen the situation, microenvironment of the tumor itself, in fact, is another formidable feature that escalates the complexity of cancer dynamic (8, 15, 16).

**Figure 1:**
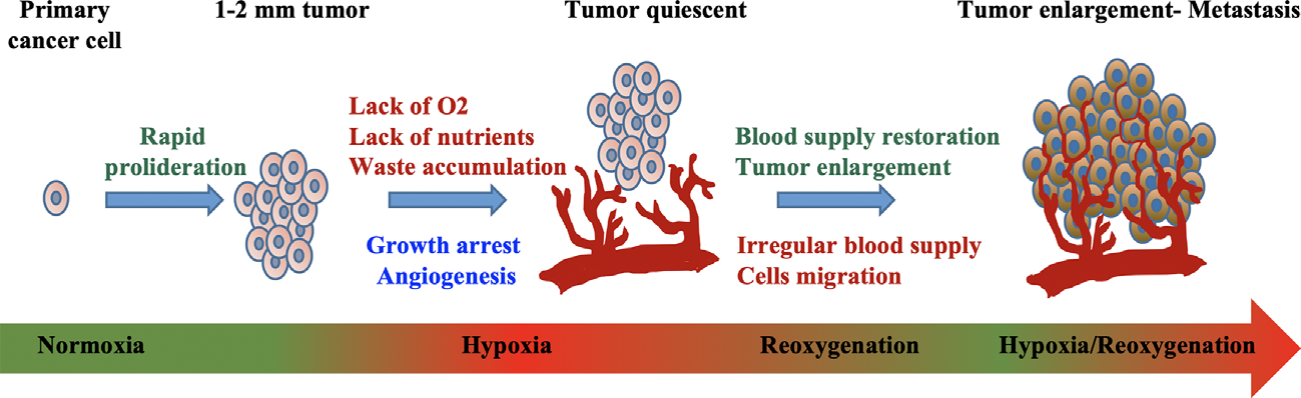
Mechanism of hypoxia-induced tumor development. Exponential cellular proliferation results in the formation of tumor. Impenetrable blood vessel that supplies oxygen and nutrients causes hypoxic environment where waste accumulates and growth arrest. Tumor cell adaptation such as the alteration in cellular metabolism, angiogenesis, vasculogenesis, and metastasis lead to irregular blood supply, all of which further promote tumor progression. Modified from “Quantitative profiling of chromatome dynamics reveals a novel role for HP1BP3 in hypoxia-induced oncogenesis,” by B. Dutta et al. (19), Molecular & Cellular Proteomics, 13(12), p. 3236–3249. Copyright 2014 by the American Society for Biochemistry and Molecular Biology, Inc. Reprinted with permission.

In addition to the complex dynamic interaction between tumor and stromal cells in the tumor microenvironment, changes in the availability of oxygen has been found to cause alteration in epigenomic, genomic, and proteomic profiles in cancer cells. In normal physiological condition, the oxygen tension of the cell environment is between 5 and 10%, whereas hypoxia is a condition in which there is a deprivation in oxygen level around 1–2% or below (17). The role of hypoxia in carcinogenesis and therapy resistance has been recognized for at least half a century (18). At the initial phase of solid tumor growth, the microenvironment of tumors with a diameter beyond 1 mm become hypoxic due to inadequate oxygen supply (**Figure 1**) (19).

**Figure 2:**
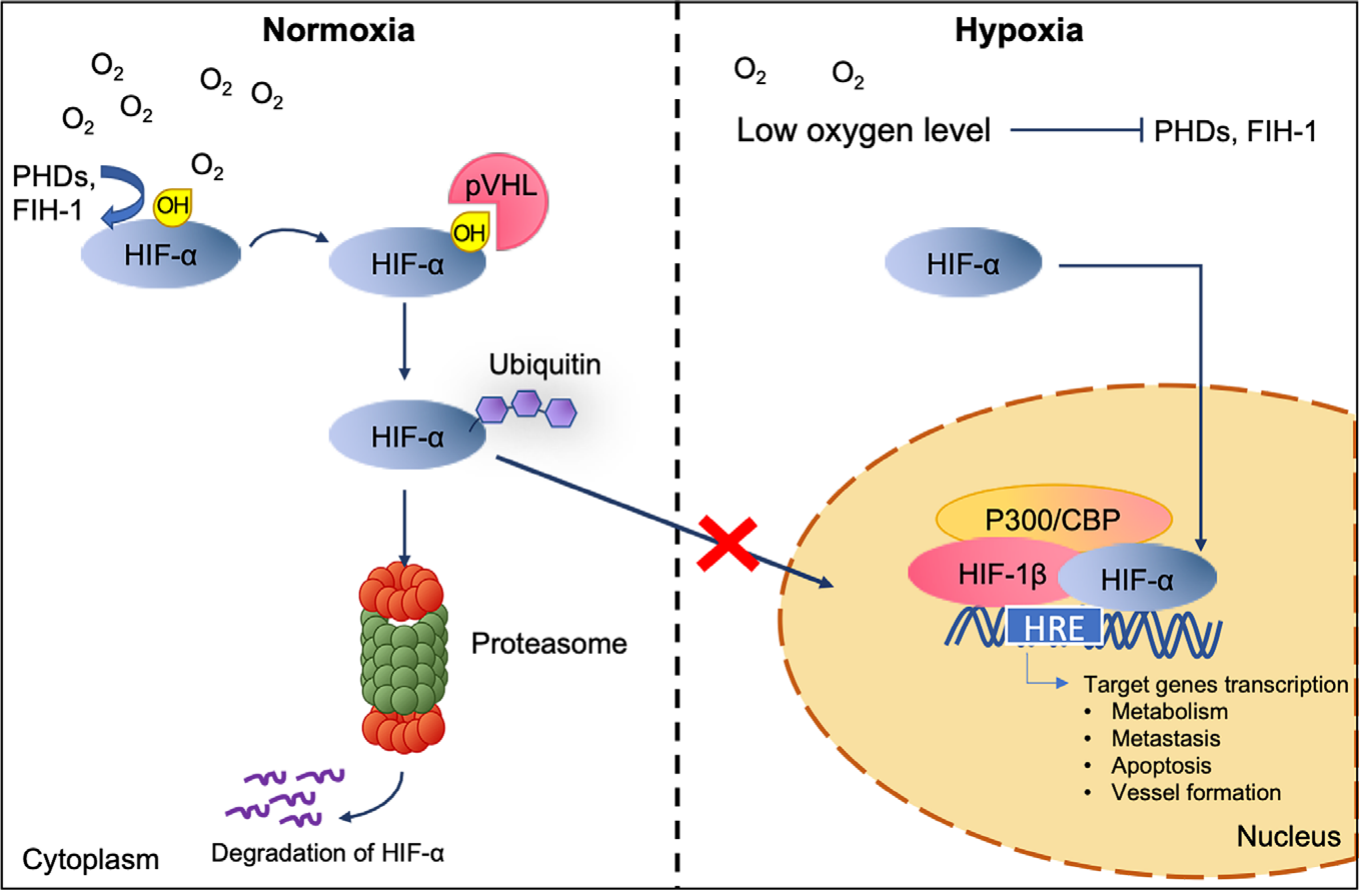
HIF interaction and degradation pathway in normoxic and hypoxic conditions. The level of HIF-*α* is maintained by the recognition of the von-Hippel Lindau tumor suppressor (pVHL) to the hydroxylated HIF-*α* subunit for ubiquitylation and subsequently proteasomal degradation under normoxic. Under low oxygen level, untargeted HIF-*α* subunits accumulate and translocate to the nucleus. Once inside, dimerization of HIF-α with HIF-1*β* and its co-activators p300/CBP activate the target genes transcription (20).

Under hypoxic conditions, tumor cells undergo specific gene translational changes, following the intracellular signaling pathways alteration. One of the well-studied modulators of the cellular response to hypoxia is the hypoxia-inducible factor (HIF) transcription factor family (20–23) (**Figure 2**). HIF contains a heterodimer of an oxygen labile subunit HIF-α and an oxygen-insensitive HIF-1*β* (24). In normoxia, HIF-*α* are hydroxylated by Prolyl-Hydroxylase (PHD) and Factor Inhibiting HIF (FIH-1), oxygen sensor enzymes, leading to rapid proteasomal degradation of HIF-*α* subunits. Contrarily, the deprivation of oxygen will lead to the accumulation of HIF-*α* and the translocation into the nucleus, resulting in the activation of genes involved in adaptive cellular changes as a response to low oxygen stress (25). It is noted that HIF mediated gene expression can also be achieved by hypoxia-independent activation of reactive oxygen species, nitric oxide, cytokines, G protein-coupled receptors, toll-like receptors and alarmins receptors, involving pathways such as PI3K/AKT/mTOR (26, 27), NF-*k*β (28), p38 and ERK (29). As a result, these pathways are shown to regulate cells apoptosis (*via* BNIP-3 and p53), metabolism (*via* GLUT-1 and GSK), metastasis (*via* EMT, CXCR4, E-cad, CAIX, LOX, and MMPs) and vessel formation (via VEGF, SDF-1, Ang-2, MMPs) (17). Such changes as a response to hypoxia have been demonstrated to ultimately drive tumor progression, enhance aggression, and promote metastatic phenotypes (15, 17).

In addition, it is worth to clarify that an induction of HIF pathway with resultant angiogenesis does not lead to normoxia recovery (30). Although the newly formed vessels can provide rapid-growing cancer cells with nutrients and oxygen for survival, the hypoxia-induced neovasculogenesis often results in an abnormal and leaky vascular network, causing irregular and sluggish blood flow (**Figure 1**). Consequently, this dysfunctional vascularization will eventually fail to provide enough blood supply for the fast-expanding cancer population (31), and vicious cycle of hypoxia condition begins (32).

As previously discussed, hypoxia can establish new cell phenotypes through induction of various signaling pathways, affecting clinical responses to therapy. Tumor hypoxia has been demonstrated to increase the expression of anti-apoptotic Bcl-2 and Bcl-xL and decrease pro-apoptotic Bcl-2 such as Bax, Bad and Bid. Failure to activate stress-mediated apoptosis in tumor hypoxia ‘primed’ tumor cells to become resistant to cancer treatment (33, 34). Similarly, hypoxia-induced autophagy activation or cell cycle arrest at G1 phase in cancer cells as an adaptation to stress lead to cell survival under cancer therapy (34). The resistance of cancer cells to treatment has challenged the medical industry as many conventional drugs and radiotherapy aim to induce cancer cells apoptosis *via* endogenous mechanisms (35, 36). Example of current drugs use in cancer that have shown to be less effective in hypoxic regions in tumors are 5-Fluorouracil (37), Actinomycin D (37–39), Sorafenib (40) and Bleomycin (37–39).

Undoubtedly, hypoxic regions in tumors have become one of the mechanistic conduits that promote cancer progression. However, tumor hypoxia is exploitable in cancer treatment. For instance, disrupting molecular mechanisms underlying hypoxia-induced adaptation may be the key to the future development of effective therapies (41, 42). In fact, hypoxia-selective cytotoxins and drugs are emerging as a new strategy to treat different types of cancer (43). Examples of drugs that directly inhibits HIF activity are the well-established rapamycin (inhibits mRNA/protein expression) (44), Aminoflavone (inhibits mRNA/protein expression (45), Acriflavine (inhibits HIF-*α*/HIF-1*β* dimerization) (46) and Bortezomib (inhibits transcriptional activity) (47). Furthermore, hypoxia prodrugs that are inactive in normoxic but are toxic under hypoxic condition have been developed to selectively target hypoxic tumor cells (48). However, the results of many of these drugs in preclinical and clinical trials do not show maximum therapeutic effect. This may be due to the lack of ‘personalized’ and tumor-specific target (43). In this respect, discovering potential hypoxic-associated tumor development pathways may lead to a successful hypoxia-targeted therapy.

## Mass Spectrometry-Based Proteomics Approaches for Hypoxia-Associated Cancer Research

Mass spectrometry (MS) for proteomics is a powerful analytical technique that has the potential to revolutionize biomedical research. The development of different MS ionization techniques [electronspray ionization; ESI (49) and matrix-assisted laser desorption/ionization; MALDI (50)], mass analyzers (quadrupole; quadruple ion trap, QIT/linear ion trap, LIT or LTQ; time-of-flight, TOF; Fourier-transform ion cyclotron resonance, FTICR, and Orbitrap) (51) and fragmentation methods in tandem mass spectrometry (MS/MS) [collision-activated dissociation, CAD (52)/collision-induced dissociation, CID (53); electron ionization dissociation, EID (54); electron capture dissociation (55, 56)/electron-transfer dissociation, ETD (57)] has presented an increasing power of both protein identification and analysis at different dimensions (*e.g.* protein expression, structure, interaction, modification, *etc.*); and therefore, allows greater understanding of complex biological processes and diseases (58–60).

Like many other research fields, cancer hypoxia has greatly benefited from its recent development in MS-based proteomics for the identification of key players and the underlying pathological mechanisms through protein profiling in complex biological samples. In such studies, differential proteomes across various types of cells/tissue/clinical samples in association of hypoxic conditions are analyzed. Proteins that are repeatedly identified across different samples suggest their likelihood of having an active role in hypoxia regulation and thus may lead to the generation of novel targeted cancer therapies (**Table 1**) (76, 77). On the other hand, a protein (or a set of proteins) of high confidence that is (are) exclusively identified in specific samples could act as candidate biomarkers for clinical use (78–81). In the following sections, we collectively describe the successful application of advance quantitative proteomics approaches coupled with different MS analytical platforms and experimental strategies which have been undertaken to overcome present limitations, focusing on studies published within the last ten years (82).This will allow us to gain valuable insights for meaningful translational research in the field of hypoxia-associated cancer pathology.

**Table 1:** Comprehensive list of the potential therapeutic targets from labeled and/or Targeted quantitative proteomic approach in hypoxic cancer cells.

Method	Potential biomarkers for cancer diagnosis	Cell line	Sample type	Reference
Metabolic labeling				
SILAC	GM3 synthase	HeLa	WCL	(61)
	Subunit B of respiratory complex II			
	Formin-2	NARF2	WCL	(62)
	Retinoic acid-induced protein 3	SW620	WCL	(63)
	Histone demethylase jumonji domain containing protein 2C	HeLa	WCL-pulldown	(64)
	Lysyl oxidase	MDA-MB-231	Secretome	(65)
pSILAC	PHD finger protein 14	A431	WCL	(66)
	Endoplasmic reticulum disulphide oxidase 1α	MIAPaCa-2	WCL	(67)
	Hypoxia-induced angiogenesis regulator	NCBP2-AS2	Secretome	(68)
Chemical labeling				
iTRAQ	Ku70/Ku80 dimer	A431	WCL	(69)
	Integrin *α*3	A431	WCL	(70)
	Calmodulin-1	A431	WCL	(71)
	Calumenin			
	Reticulocalbin-1			
	Heterochromatin protein 1-binding protein 3	A431	Chromatome	(19, 72)
TMT	Vitamin B_12_ transporter protein TCN2	U87	WCL	(73)
	Colony stimulating factor-1	B16-F0	Exosome	(74)
	Ferritin heavy/light chain			
	Lysyl oxidase	U87	Exosome	(75)
	A disintegrin and metalloproteinase with thrombospondin motifs 1			
	Vascular endothelial growth factor A			
Targeted				
MRM	Ku70/Ku80 dimer	A431	WCL	(69)

WCL, Whole cell lysate.

## Advance MS-Based Strategies

**Figure 3:**
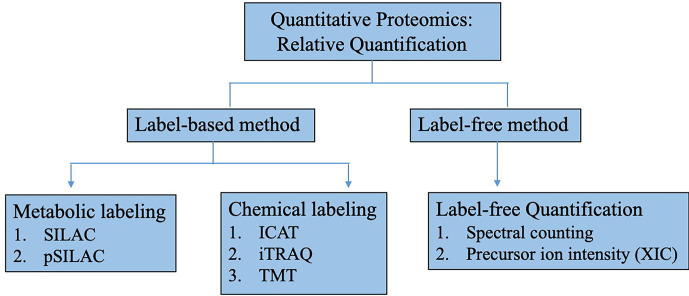
Methods for relative quantitative proteomics research. Relative quantitative proteomics can be classified into label-based or label free methodologies. In label-based method, labeling can be done by metabolically incorporating stable heavy isotope into proteins such as in stable isotope labeling with amino acids in cell culture (SILAC). Protein/peptides can also be chemically labeled with isotopic tag after sample preparation such as isotope-code affinity tag (ICAT), isobaric tags for relative and absolute quantitation (iTRAQ) and tandem mass tags (TMT). For label-free method, quantification is done by tandem mass spectral counting or MS precursor ion intensity through extracted ion chromatogram (XIC).

Although a multitude of hypoxia mediators and biomarkers have been identified and quantified using label-free relative quantification, there has been a remarkable transition from static proteome analysis to elucidate diverse aspects of the dynamic proteome through new techniques such as mass spectrometry imaging (MSI) for spatial distribution of molecules (83) or multiple reaction monitoring (MRM) and stable heavy isotope labeling combined with MS for targeted (84) and absolute quantitation (85). The different uses of advance multiplexed quantitative techniques, whether carried out independently or in combination, have enabled investigators to quantify precise changes in protein/peptide and PTM abundances in multiple biological matrices simultaneously within a single MS analysis (86–88). A summary of quantitative proteomic analysis is depicted in **Figure 3**. The improvement in robustness, resolution, and specificity has proven useful especially in the identification of true protein interactions (89, 90), detecting the presence of specific subset of proteins/peptides in highly complex samples (biomarkers) (91, 92) and defining accurate relationship between mechanism of signaling pathway and its downstream biological responses (93, 94).

### Metabolic Labeling Coupled With MS

Metabolic labeling with ^15^N is the very first isotope-based labeling technique to be applied for quantitative proteomic analysis (95). After the incorporation of isotopes to live cells through growth medium, the acquired samples can be combined prior to cell lysis, thus minimizing errors that may arise during subsequent multi-step proteomics sample preparation from purification to fractionation steps before MS analysis. The introduction of mass shift to sample peptides with the labeled heavy isotype of nitrogen allows relative quantification unaffected by sample purity or instrument response when comparing spectral intensities of heavy peptide to that of naturally occurring light peptide (^14^N) in control sample (96, 97). However, this method limits the analysis to two samples per experiment and it is not suitable for organisms with long generation times, and high ^15^N enriched isotope (>99%) is necessary for less dispersive peptide isotope distribution and accurate quantitation. The later developed method of stable isotope labeling by amino acids in cell culture (SILAC) for whole-proteome MS analysis of multiple samples (up to five) in different conditions has become a popular alternative approach. In this technique, only selected amino acids are being labeled. Typically, arginine and lysine are selected since the subsequence proteolytic cleavage with trypsin during proteomics workflow will result in a pool of peptides that contain at least one labeled amino acid for downstream quantification by MS (96, 98). It is noted that although metabolic labeling requires living cells, recent studies have expanded the use of SILAC to tissue analysis using super-SILAC approach (99–101).

#### Application of SILAC Labeling and LC−MS/MS for Quantitative Proteome Analysis of Hypoxia-Associated Proteins *In Vitro*

The proteomic analysis based on SILAC and high resolution tandem mass spectrometry such as Orbitrap mass spectrometer is often the method of choice to identify and quantify cancer hypoxia-regulated proteins from complex peptide mixtures. One important work by Yates et al. has demonstrated that the combination of the high-throughput, the high-resolution and high-accuracy capabilities of the Orbitrap mass analyzer enabled four- to five-fold improvements in the number and quality of the peptide ratio measurements compared to low resolution LTQ (102). Using this method, many studies have successfully characterized novel hypoxia-perturbed pathways and mediators in various cell types of human system, including breast cancer (103), cervical carcinoma (HeLa cells) (61, 104), osteosarcoma (62), colorectal tumor (63).

SILAC-based LC−MS/MS approach enabled researchers to descriptively explain the global changes and adaptation in tumor cells under oxygen deprivation (103, 104). In 2014, a comprehensive proteome analysis was conducted on 4T1 cells of breast cancer model to monitor the ratio of differentially regulated proteins under hypoxic conditions (103). SILAC in conjunction with LTQ-Orbitrap has identified a total of 131 proteins with significant quantitative changes, 60 of which were upregulated (*e.g.* glyceraldehyde-3-phosphate dehydrogenase, L-lactate dehydrogenase A chain, and phosphoglycerate kinase) and 71 downregulated (*e.g.* ribosomal proteins and ubiquitin-associated proteins). The classification of altered proteins according to biological process and molecular functions using PANTHER bioinformatic tool (www.pantherdb.org) revealed that hypoxia-associated proteins are mainly involved in metabolic processes, cellular process, binding, and catalytic activities.

In line with proteomic study on breast cancer model, another quantitative proteome analysis conducted by Bousquet et al. in 2015 using HeLa cells as a representative of human cancer system has revealed some similar findings (104). In this study, 125 unique hypoxia-regulated proteins were identified. 72 proteins that were found upregulated are mostly proteins of glycolysis (noted that lactate dehydrogenase A, phosphoglycerate kinase 1 and 2 were also identified; Supplementary Table 1). However, interestingly, most of the 53 downregulated proteins were identified as mitochondrial proteins (*e.g.* mitochondrial ribosomal proteins and mitochondrial translocases—these proteins have not previously been reported as hypoxia-regulated proteins in tumor cells) and a few were citric acid cycle (CAC) related proteins. Again, Gene Ontology based analysis by DAVID *(* http://david.abcc.ncifcrf.gov/) and PANTHER also revealed that the main biological process affected by hypoxia was metabolism, and that proteins with catalytic and binding activities were shown to dominate the molecular function. Collectively, the upregulation of various glycolytic enzymes, particularly, lactate dehydrogenase, which function to convert pyruvate to lactate, and the downregulation of mitochondrial proteins suggested metabolic shifts of cancer cells from cellular respiration to inefficient glycolysis for ATP needs *via* enhancement of glycolysis and suppression of cellular respiration in the absence of oxygen. Together, the change in metabolic pathways-related proteins under hypoxia condition supports the key difference between normal tissue and cancer metabolism which was first noted since 1920s. These cancer cells undergo a phenomenon termed the Warburg Effect, shifting from oxidative phosphorylation to aerobic glycolysis despite the availability of oxygen (105). In addition, Bousquet et al. (2018) expanded the use of his latter quantitative proteomic data to explain the potential mechanism of N-glycolyl (NeuGc) GM3, a tumor-associated antigen, generation in human cancer cell line induced by hypoxia (61). Specifically, GM3 synthase and subunit B of respiratory complex II (SDHB) that were found upregulated under hypoxia condition may be responsible for the increase in NeuGc incorporation into human cancer cells. Despite the two proteins being potential targets for cancer therapy, further validation of the hypothesis is needed.

Several novel mediators of the hypoxia-induced adaptive response that function to protect cancer cell from apoptosis were successfully identified by SILAC and Orbitrap. In 2013, quantitative analysis of nucleolar proteome alteration following induction of p14ARF, a tumor suppressor gene, was performed on NARF2 osteosarcoma cells (62). The MS analysis identified relative expression ratio of more than thousands of nucleolar proteins, and that Formin-2 (FMN2) was highly induced by ARF. Subsequence analysis using qPCR confirmed that FMN2 expression can also be induced by hypoxia (%1 O_2_), and the depletion of FMN2 resulted in apoptosis induction. Similarly, the most recent study conducted by Greenhough et al. in 2018 reported the upregulation of GPRC5A in colorectal tumor cells grown under hypoxia stress (63) despite unchanged levels found in previous omics studies on HeLa cells (SILAC and Orbitrap) (104) and breast cancer cells (SILAC and Orbitrap based LC-MS/MS and MALDI-MSI) (103). This suggested that GPRC5A could be a colorectal cancer-specific hypoxia mediator. Functional analysis of GPRC5A confirmed that GPRC5A promoted hypoxic cancer cells survival *via* the Hippo pathway effector YAP. Collectively, these data highlight FMN2 and GPRC5A as targets for cellular vulnerabilities of cancer cells.

An important co-activator of hypoxia-inducible factor 1*α* (HIF-1*α*) was verified using pull-down of SILAC labeled targets coupled with the high-performance quadruple time-of-flight (Q-TOF) mass spectrometer (64). As previously mentioned, hypoxia-inducible factors (HIFs) are activated in solid tumors as a result of hypoxia exposure. The activation of HIF in turn targets genes encoding proteins that are critical in cancer progression (17). In this study, endogenous HIF-1*α* and HIF-2*α*-interacting proteins in HeLa cells treated with prolyl hydroxylase inhibitor, dimethyloxalylglycine (DMOG), to induce HIF transcriptional activity were screened using GST fusion protein containing protein binding domain of HIF-1*α* (residues 531–826) or HIF-2*α* (450–870) as bait for pulling down interacting partners using quantitative isotope labeling by SILAC. MS identified a total of 146 proteins as HIF-1*α* and HIF-2*α*-interacting proteins, with 44 proteins as specific HIF-1*α*-interacting partners and 42 proteins as specific HIF-2*α*-interacting partners. The ratio of peak intensities obtained from HIF-1*α* (heavy peaks) or HIF-2*α* (medium peaks) must be ≥2 than the peak intensities obtained from control (light peaks) to be considered quantitatively significant. Out of 44 specific HIF-1*α*-interacting partners, the histone demethylase jumonji domain containing protein 2C (JMJD2C) encoded by KDM4C gene, which has been previously reported to be HIF-1 target gene, was further investigated. Experimental validation by JMJD2C knockdown has proven the significance of JMJD2C in breast tumor growth and metastasis to the lungs of mice.

SILAC-based approach has been applied in proteomic profiling of secretomes. Proteins secreted from cells will be labeled with “heavy” amino acids, which can then be separated from contamination of “light” amino acids of proteins from fetal bovine serum in the cell culture media (106). As noted previously that metabolic labeling permits mixing of different samples prior to quantitation, SILAC is thus suitable for secretome research as any errors caused by extensive sample processing in isolation and enrichment steps to obtain secretome will be eliminated. In 2015, a study conducted by Erler et al. serves as a good experimental model for hypoxia cancer secretome quantitative analysis (Quadrupole-Orbitrap LC-MS/MS system) (65). This selective sorting procedures of secretome with sophisticated MS instrumentation has provided accurate global identification and quantitation of secreted proteins that are uniquely release under different breast cancer cell types and hypoxic conditions (*i.e.* MDA-MB-231 parental 21% and 1% O_2_, MDA-MB-231 Bone-Tropic 21 and 1% O_2_). In this study, lysyl oxidase (LOX) was identified as one of most highly upregulated (>2.25-fold) secreted proteins associated with osteotropism in hypoxia-induced secretome release from MDA-MB-231 Bone Tropic breast cancer cell lines (107). Subsequence functional analysis *in vivo* successfully identified secreted LOX as a novel hypoxia mediator that initiates the formation of pre-metastatic bone lesion which eventually allows circulating tumor cells to colonize the bone (65).

#### Application of Pulsed-SILAC Labeling and LC−MS/MS for Quantitative Analysis of the Dynamic State of Hypoxia-Associated Proteins Turnover

The conventional SILAC labeling provides the knowledge of a steady-state amount of protein in the cell. Nonetheless, it is important to emphasize that the concentration of protein found does not always correlate with rate of protein synthesis, but rather the result of the balance between protein synthesis and degradation (108). For this reason, understanding dynamic proteome relationship is important when interpreting proteins abundance data from proteomic experiments to understand how cells regulate their proteomes to execute numerous cellular responses to particular stimuli.

The emergent approach to identify *de novo* synthesis of proteins and the measurement of their half-life by adapting the classic pulse-chase experiment to SILAC workflows (pSILAC) has enabled researchers to gain insights into the dynamics of protein expression on a proteome-wide scale. As a modified version of the conventional SILAC approach, instead of mixing normal light and heavy peptide samples after isotopes incorporation, pSILAC involves the switching of amino acid in the media from normal light to heavy (or *vice versa*) at a certain time point prior to perturbation such as hypoxia stress. At this time point on, proteins translating in cells are pulse-labeled and all newly synthesized proteins are distinguishable from the preexisting proteome. Then, the relative abundance of light and heavy peptides overtime can be assessed to quantify the actively translating proteins induced by the perturbation.

It was not until recently that quantitative pSILAC was first applied to study proteome in hypoxia cancer model. In 2016, a study was performed on U87MG human glioblastoma and 786-O human renal clear cell carcinoma cell lines to investigate the effect of oxygen tension on translation efficiency. The results revealed that oxygen perturbation can cause widespread changes in protein output, with minimal changes on mRNA levels. Notably, this study reported that HIF target genes show no alteration upon oxygen deprivation in contrast to an increase in translation level (109).

Later on, a global analysis of newly translated proteins between normoxic and hypoxic (24 h) A431 epidermoid carcinoma cells revealed an intriguing finding that only 5% of total proteins were upregulated, and more than 60% were translationally suppressed in hypoxia condition (66). In concordance with the previously mentioned study by Bousquet et al. [in 2015 (104) and 2018 (61)] using the traditional SILAC method, key modulators of aerobic glycolysis [*i.e.* glucose transporter 1 (GLUT1) and hexokinase 2 (HK2)] were found *de novo* synthesized for up to 1.8-fold, and that protein synthesis of key TCA cycle enzymes were suppressed. This data confirmed the metabolic switch from oxidative phosphorylation to glycolysis. However, in this latter pSILAC study, some glycolytic enzymes including HK1 and lactate dehydrogenase A that were previously found upregulated are suppressed (66). The possible explanation to this phenomenon is a slow rate of protein degradation, which resulted in the overall increase in this set of glycolysis proteins in hypoxic cells (110). Moreover, information from pSILAC proteomic analysis reveal for the first time that PHF14 is an epigenetic modulator that plays a key role in cell growth cessation, a classical cellular response in cells under hypoxia (66).

In a pancreatic cancer model, pSILAC and high resolution MS were used to identify the dynamic expression of hypoxia-induced mediators. In this study, the elevated expression of proteins that play a role in immune suppression, angiogenesis, metabolic activity, and metastasis were found. The increase in this set of proteins is associated with poor patient survival in various stages of pancreatic cancer (67). Interestingly, one of the highly inducible proteins upon hypoxia stress, ERO1α, was demonstrated by genetic deletion of ERO1*α* gene in pancreatic cancer cell lines and mouse xenograft model to slow down growth rate of tumor cells, indicating the significance of ERO1*α* in cancer progression *in vivo* and *in vitro* (67).

It is known that cancer-associated fibroblasts (CAFs) play an important role in the pathogenesis of cancer, but the response of CAFs upon oxygen restriction remains unclear. In 2019, Kugeratski et al. performed conventional SILAC-MS to characterize both proteome and secretome of hypoxic CAFs. In this study, the function of NCBP2-AS2 was highlighted as its level increased significantly in hypoxia condition. Subsequent analysis using a pSILAC-based workflow confirmed that hypoxia induces NCBP2-AS2 posttranscriptionally by enhancing its translation. Functional analysis of NCBP2-AS2 confirmed the pro-angiogenic and pro-migratory function of CAFs under hypoxic condition (68).

### Chemical Labeling Coupled With MS

While the original metabolic labeling is not applicable in samples that are not metabolically active (*i.e.* biofluids and biopsies material), the introduction of isotope labeling to proteins or peptides using chemical reactions such as isotopic isotope-coded affinity tag (ICAT) (111) and isotope-coded protein labels (ICPL) (112) or isobaric tag for relation and absolute quantification (iTRAQ) (113) and tandem mass tags (TMT) (114) labeling can be done on any proteome sample (115). Moreover, chemical labeling has been reported to reduce sample processing time (116). However, major limitations include the requirement of specific proteins (*i.e.* cysteine or lysine) to quantify and chemical labeling is performed at the late stage of sample preparation, which therefore increases the chance of errors being introduced in the multi-steps proteomic sample preparation (115). Using the chemical labeling approach, a number of studies have successfully uncovered potential cancer biomarkers as well as key players in tumorigenicity and resistance to cancer therapy.

Isobaric tag-based high-throughput quantitative proteomics is based on the covalent conjugation of the stable isotope to the N-terminus and side chain amines of the peptides. The individual samples labeled with different isobaric tags are often pooled and subjected to fractionation by liquid chromatography prior to LC-MS/MS analysis. Despite the superior quantitation accuracy of SILAC, isobaric tag methods outperforms the method of SILAC in terms of sensitivity with a capacity to detect reporter ions in the low *m/z* region and the ability to be multiplexed to up to 8 (for iTRAQ) and 16 (for TMT) samples being processed simultaneously.

#### Application of iTRAQ for Quantitative Analysis of Multiplexed Hypoxia Samples

The first study that incorporated the use of iTRAQ for relative quantification in hypoxia and cancer model was in 2010 (117). Park and colleagues performed iTRAQ labeling and LC-MS/MS on the secretome of hypoxic A431 squamous carcinoma cells to elucidate the tumorigenic mechanisms (117). Isobaric tags of varying mass were labeled on cells under normoxic, hypoxic 48 h, hypoxic 72 h, and 48 h hypoxic followed by 24 h reoxygenation conditions. The levels of protein expression were then quantitated using relative peak intensities of the tags. Using this approach, the results revealed significant alteration in secretome including soluble proteins and exosomes/extracellular vesicles of A431 cells under hypoxia condition which were linked to their metastatic and angiogenic potential. Notably, there was a decline in the secretion of extracellular matrix protein components that involved in focal adhesion, antiangiogenic factors, and regulator of intercellular adhesion; and an increase in potent pro-metastatic factors. This data supported the observed phenomenon that under hypoxia condition, the tumor cells exhibited reduced adhesion and enhanced invasiveness as determined by adhesion and chemoinvasion and induced angiogenesis by chorioallantoic membrane (CAM) assay.

With the same A431 cells, the differential protein expression after hypoxia and reoxygenation treatment were investigated using iTRAQ quantitative method (2013) (69). More than 4,000 proteins were identified, 1200 of which were modulated >1.2 fold. Several potential novel targets for tumor therapy were identified. This includes proteins that are involved in glycolysis pathway, STAT1 pathway, glycoprotein synthesis, and integrin (69, 70). Notably, the upregulation of non-homologous end-joining pathway which plays role in DNA repair in hypoxic A431 cells were quantified. iTRAQ labeling has also been used to identify radiotherapy-resistant protein targets. Quantification of the change in proteome of irradiated hypoxic A431 cells by low-dose *γ* irradiation showed a significant upregulation of several calcium binding proteins (71). Subsequent knockdown using shRNA of genes encoding these proteins resulted in hypoxic A431 cells which were resistant to radiation. These findings have, for the first time, identified radiotherapy-resistant protein targets for cancer patients undergoing radiotherapy.

The robust and sensitive iTRAQ quantification has quickly become a popular method to quantify sub-cellular fractionation proteins (118). More recently, iTRAQ labeling has been used to compare the profile of chromatome, chromatin-associated proteins, in normoxic, hypoxic and re-oxygenated A431 cells (19). Chromatin is a complex structure containing both DNA and proteins which function to regulate cellular processes that requires access to DNA (*i.e.* DNA replication, DNA transcription, DNA-repair). Although it is known that the transcriptional change during hypoxia is mainly regulated by the HIF, emerging evidence has shown that gene transcriptional changes exerted by other non-dependent chromatome-induced epigenetic changes also play a key part of the hypoxia response (25, 119, 120). Therefore, identification of chromatin-associated proteins is essential in understanding the cellular response to hypoxia environment. The iTRAQ method for quantitation of differentially regulated chromatome has shown that 819 proteins changed their chromatin association topology under hypoxic conditions. Functional investigation of the chromatin organizer protein HP1BP3 has revealed its role in regulating chromatin condensation upon hypoxia treatment, which can mediate tumor progression and acquire therapy-resistant traits (19). Furthermore, iTRAQ-based proteomics to quantify chromatin-associated proteins during the interphase of cell cycle has again identified HP1BP3 to be a key player that control the cells ability to proliferate (72).

#### Application of TMT for Quantitative Analysis of Hypoxic Cancer Cells Proteome/Secretome

In 2017, TMT was utilized along with label-free LC-MS/MS to analyze the hypoxic and normoxic proteome of U87 glioblastoma cells (73). Zhang et al. adapted the SILAC technique to TMT and label-free proteomics in replicating cells by first incorporating light arginine to the proteins instead of the heavy isotope-containing arginine for 5 days, followed by TMT stable isotope labeling. This way, not only did the expression of newly synthesized proteins can be quantified, but the cell proliferation rates can also be monitored. The proteins quantified by TMT proteomics revealed that hypoxia upregulates glucose transport and glycolysis pathways. From the proteomic data, glucose transporter (GLUT1), DUSP4/MKP2, and RelA proteins which are involved in inflammation, enzymes for glycolytic pathway, and proteins involved in cells transition from an epithelial phenotype to a type III EMT mesenchymal phenotype were elevated. In addition, a novel finding was found that under hypoxic condition, the vitamin B_12_ transporter protein TCN2 is significantly downregulated, resulting in cells’ growth arrested and also plays an important role in directing cancer cell transformation toward the highly aggressive mesenchymal/CSC stage.

TMT 6-plex tagging of three independent sets of normoxic and hypoxic exosomal protein released from glioblastoma (GBM) cells was performed by Kora et al. in 2018 to elucidate the mechanisms of cancer progression (75). The results revealed that hypoxia condition significantly induced the upregulation of various exosomal proteins that were reported to be involved in tumor progression, metastasis and angiogenesis. In another study, TMT-based analysis was performed on exosome isolated from B16-F0 mouse melanoma cell culture supernatants of normoxic and hypoxic conditions (74). The result showed an enrichment of proteins that are associated with immune responses. Concomitant with MS result, the isolated exosomes has been demonstrated to influence the macrophage M2-like polarization and promote oxidative phosphorylation in bone marrow-derived macrophages *via* transfer for let-7a miRNA, which resulted in the inhibition of the insulin–Akt–mTOR pathway. These findings provided multiple targets including the signaling pathway and proteins that contribute to cancer metastasis and that hypoxia-induced exosomal release from tumor cells may be a source of potential biomarkers for cancer diagnosis (121).

### Multiple Reaction Monitoring

MRM is a sensitive and highly specific MS technique that has been extensively used for the quantification of small molecules (87, 115), for example, the metabolite profiling of clinical samples (122). In typical MRM experiments, the target precursor ions are fragmented and only the pre-selected fragment ions of interest are being monitored (123). Likewise, the same principle of quantification is applicable to proteomic applications. MRM is one of the most commonly used technique in targeted proteomic assays. This specific selective quantification of peptide of interest provide researchers with a tremendous advantage especially in verifying proteomic markers when specific antibodies for immune assay are not available.

The use of MRM has also been applied for hypoxia and cancer analysis. As discussed previously, proteome change identified by iTRAQ labeling coupled with LC-MS/MS of A431 epithelial carcinoma cells induced by hypoxia and reoxygenation revealed a set of proteins perturbed by hypoxia and not in normoxia (69). This set of unique protein expression was subsequently confirmed using the LC-MS/MS-MRM quantification method, in which most of the proteins are verified. Notably, upregulation of key proteins in the nonhomologous end-joining pathway, Ku70/Ku80 dimer, was reported to be much higher than the quantification result obtained from iTRAQ (ratio 2.05 vs. 1.1–1.2, respectively). In this specific experimental setting, this finding demonstrated the superior sensitivity of MRM method compared to iTRAQ labeling.

## Label-Free MS-Based Strategies

With the advancement in MS, the use of label-free quantitation has increased over the past few years. Numerous studies demonstrated the success in using label-free method for relative protein quantitation to investigate how hypoxia drives cancer progression (73, 124–127). Label-free MS-based protein quantification is commonly used in proteomic analysis. The major advantage in using label-free quantitation is that it requires no labeling of the sample and applicable to all types of proteomic samples. Two of the most widely performed methods are spectral counting and peptide peak intensity measurement. Spectral counting is more suitable for proteins with high number and high abundance in the samples. Only sufficient amounts of peptides to trigger MS/MS spectra are quantifiable. Therefore, spectral counting is less reliable for low mass and low abundant proteins (128). On the other hand, quantitation of proteins by peptide peak intensity compares the total amount of peptide ion intensities using extracted ion chromatogram in a specific retention time computed and showed as the area under the curve. However, it is reported that up to 40% of data variation at the peptide level can occur since the coverage of common ions are strongly dependent on the amount of sample loaded, column condition, and calibration. Thus, achieving reproducibility is difficult between different sample runs (129).

## Concluding Remarks

The field of proteomics is based on the analysis of large number of proteins simultaneously. The use of MS is a powerful technology that facilitates the success of quantitative and differential proteome analysis. The number of publications on hypoxia and cancer is escalating with increasing in knowledge in this topic. Choosing the right technique depends on research questions being addressed as one methodology may be better than another for one’s particular experiment. It is necessary for scientists to be able to keep up with the rapid advancement in new analytical methods and instrumentation as well as understanding the principles, strengths and limitations of the methods.

However, there is room for improvement in MS-based proteomics. The algorithms of peptide identification and bioinformatics computational big data analysis are two areas that can be improved. Since peptides obtained from the experimental MS/MS will be assigned to theoretical peptide sequences in a given protein database, implementation of appropriate algorithms would reduce the number of incorrect peptide matches and, therefore providing protein lists of high confidence. Cancer proteome comprises extremely complex proteoforms including mutated proteins, cancer specific PTMs, hypoxia induced proteins and PTMs etc. Bottom up approach is the commonly used method in cancer proteomic research by digesting proteins to peptides using specific protease because peptides are easier to handle and separate in LC-MS/MS analysis. However, the main disadvantage of the peptide centric bottom up approach is the protein inference problem, *i.e.* the detected peptide can be presented in multiple different proteins, leading to ambiguities in determining the proteins and their biological functions. Top down mass spectrometry which measures and sequences individual intact proteins directly without digestion can precisely characterize proteoforms including mutations and post translational modifications. Thus top down approach is an essential method for studying hypoxia cancer biology and should be further developed to precisely determine proteoforms in a cancer sample for accurate interpretation of biological functions.

Despite tremendous progress of analytical hardware, tools for biological and functional annotation of data are still in its infancy. Typically, data obtained from MS are highly complex. It needs to be emphasized that choosing software applications for downstream analysis is as equally important as sample preparation and MS analysis, considering that it determines the meaningfulness of the result and influences how one interpret the data. This is set to improve with the continuous incorporation of new technologies.

Another major point that is equally important in studying hypoxia and cancer is the experimental design to create the hypoxic environment for experimentation. It has been found that the biological consequences largely depends on the time of cells exposure to hypoxia (130). Generally with similar observing trends, cancer cells under acute hypoxia *in vitro* ranging between a few minutes to 72 h have been shown to exhibit apoptotic and metabolic adaptation leading to tumor cell survival and progression (131, 132), while cells under chronic hypoxia ranging from a few hours to many weeks have demonstrated the high frequency of DNA breaks leading to genomic instability and mutagenesis (133), both of which have been shown to increase radio-resistance in cancer (134). Nonetheless, it needs to be emphasized that these observed responses are not exclusive to acute or chronic hypoxia, and that inconsistent findings have been reported in the past (130). For this reason, standardization of the time of hypoxia exposure and the identification of the proteins underlining specific time points of treatment is important.

We have discussed the technological advancements, future directions as well as challenges in the application of mass spectrometry-based proteomics in the context of cancer and hypoxia research. With continuous improvement and newer technologies being introduced, it is very likely that there will be increased utilization of proteomics for diagnostic biomarker and therapeutic discovery in cancer.

## Author Contributions

AV drafted the manuscript. JL, KY, WC, and SS revised the manuscript. All authors contributed to the article and approved the submitted version.

## Funding

This work is in part supported by the Singapore Ministry of Education (MOE2016-T2-2-018 and MOE2018-T1-001-078), and Singapore National Medical Research Council (NMRC/OFIRG/0003/2016).

## Conflict of Interest

The authors declare that the research was conducted in the absence of any commercial or financial relationships that could be construed as a potential conflict of interest.
